# Dietary Protein, Kidney Function and Mortality: Review of the Evidence from Epidemiological Studies

**DOI:** 10.3390/nu11010196

**Published:** 2019-01-18

**Authors:** Giancarlo Bilancio, Pierpaolo Cavallo, Carolina Ciacci, Massimo Cirillo

**Affiliations:** 1 Dipartimento di Medicina, Chirurgia e Odontoiatria “Scuola Medica Salernitana”, Università di Salerno, 84081 Baronissi (SA), Italy; giancarlo.bilancio@gmail.com (G.B.); cciacci@unisa.it (C.C.); 2 Dipartimento di Fisica, Università di Salerno, 84084 Fisciano (SA), Italy; pcavallo@unisa.it; 3 Dipartimento di Sanità Pubblica, Università Federico II, 80131 Naples (NA), Italy

**Keywords:** dietary protein, kidney function, mortality, epidemiology

## Abstract

The World Health Organization recommends a minimum requirement of 0.8 g/day protein/kg ideal weight. Low protein diets are used against kidney failure progression. Efficacy and safety of these diets are uncertain. This paper reviews epidemiological studies about associations of protein intake with kidney function decline and mortality. Three studies investigated these associations; two reported data on mortality. Protein intake averaged >60 g/day and 1.2 g/day/kg ideal weight. An association of baseline protein intake with long-term kidney function decline was absent in the general population and/or persons with normal kidney function but was significantly positive in persons with below-normal kidney function. Independent of kidney function and other confounders, a J-curve relationship was found between baseline protein intake and mortality due to ≈35% mortality excess for non-cardiovascular disease in the lowest quintile of protein intake, a quintile where protein intake averaged <0.8 g/day/kg ideal weight. Altogether, epidemiological evidence suggests that, in patients with reduced kidney function, protein intakes of ≈0.8 g/d/kg ideal weight could limit kidney function decline without adding non-renal risks. Long-term lower protein intake could increase mortality. In most patients, an intake of ≈0.8 g/day/kg would represent a substantial reduction of habitual intake considering that average intake is largely higher.

## 1. Introduction

**Figure 1:**
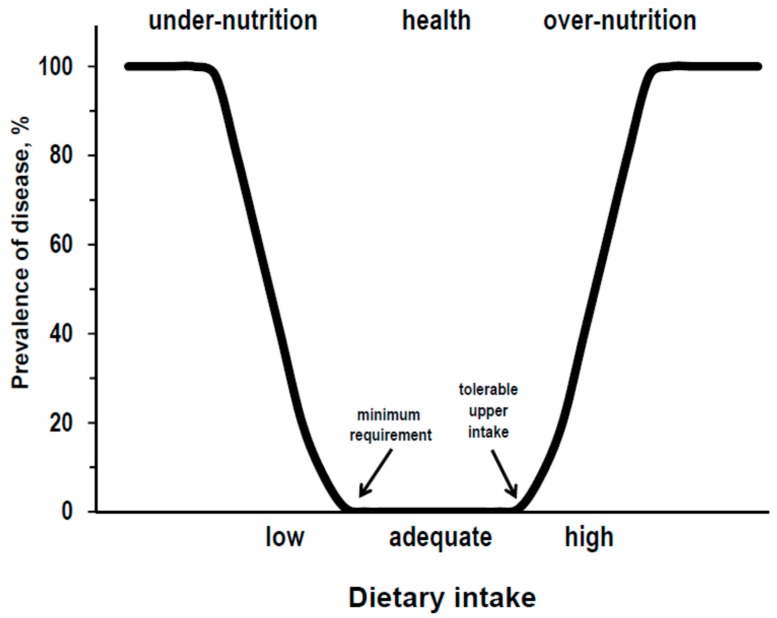
Theoretical relationship for essential nutrients between the level of dietary intake and disease prevalence.

The theoretical relationships between diet and health, for proteins as for other essential nutrients, could be described by a U-curve where intakes below the minimum requirement should associate with under-nutrition diseases, intakes between the minimum requirement and the tolerable upper limit should associate with good health, and intakes above the tolerable upper limit should associate with over-nutrition diseases (Figure 1).

In adults, the World Health Organization recommends a minimum requirement of 0.8 g/day protein per kg of ideal weight but does not suggest a tolerable upper limit [1]. The recommendation is not expressed as a percent of calories to avoid the confounding of inappropriate intake of calories. In fact, an adequate protein intake could seem low as a percent of an excessive calorie intake and, vice versa, could seem high as a percent of an insufficient calorie intake. Moreover, the normalization of the requirement for the ideal weight rather than for the actual weight is obvious for essential nutrients, such as protein, otherwise the recommended requirement should paradoxically increase in overweight individuals but it should decrease in malnourished under-weight individuals.

There is a consensus about the idea that dietary protein intake is a major physiological modulator of kidney function [2,3]. This idea is based on a number of experimental and clinical observations as proved by the fact that the words dietary protein and kidney function generates in PubMed a list of approximately 9000 publications from 1927 to 2018. One of the most consistent effects of dietary protein on kidney function is the transient up-regulation of glomerular filtration that follows the ingestion of protein-containing foods [2,3,4]. The protein-induced glomerular hyperfiltration is part of a general up-regulation of renal haemodynamics but the mechanisms underlying these effects are not well defined [2,3,4]. At a clinical level, it has been reported since the 1960s that low protein diets may slow down the rate of progression of kidney disease toward kidney failure and may improve the control of metabolic abnormalities secondary to kidney dysfunction [5]. The observations of glomerular hyperfiltration after protein intake and of favourable effects of low protein diets on kidney failure progression led to the hypothesis that the chronic or repeated induction of glomerular hyperfiltration secondary to protein intake could be one of the factors hastening the progression of kidney disease toward kidney failure [2]. 

It must be said that there are uncertainties about the long-term use of low protein diets in kidney disease, because of doubts not only for their capacity to really slow down the kidney failure progression [6,7] but also for their safety and, in particular, for the possible risk of protein malnutrition and related complications up to increased mortality risk [8,9,10]. Available data do not allay these doubts. Moreover, new studies are unlikely due to difficulties in granting and in realizing long-term controlled dietary interventions with adequate statistical power. 

Epidemiological studies investigated the possible effects of dietary protein intake with the use of a longitudinal observational design, that is analysing the incidence of outcomes among individuals with different levels of protein intake while on their own diet under free-living conditions. The present paper aims to review the results of the epidemiological studies that investigated protein intake focusing primarily on changes in kidney function over time and secondarily, if data were collected, on mortality.

## 2. Epidemiologic Studies on Dietary Protein and Kidney Function

Three epidemiologic studies reported longitudinal data on the association of protein intake with changes in kidney function over time: The Nurses’ Health study, the Prevention of Renal and Vascular End-stage Disease (PREVEND) study, and the Gubbio Study [11,12,13]. The design of all these three studies consisted of two exams: The first exam for baseline assessment of protein intake and kidney function and the second exam for the assessment of kidney function change over baseline after long-term follow-up. The Nurses’ Health study was realized in the US and enrolled female nurses from several populous US states. The cohort for investigation on protein intake and kidney function consisted of about 1600 women with ages from 42 to 68 years. The PREVEND study was done in the Netherlands and enrolled a microalbuminuria-enriched population-sample residing in the city of Groningen. The sample consisted of about 8400 individuals, both sexes, with ages from 28 to 75 years. The Gubbio study was done in Italy and enrolled a sample of the whole general population consisting of residents in the city of Gubbio, both sexes, with ages from 5 to 99 years. About 4300 adults, men and women with ages from 18 to 97 years made up the cohort for investigation on protein intake and kidney function. Duration of follow-up was approximately 11 years in the Nurses’ Health study, 7 years in the PREVEND study, and 16 years in the Gubbio study. The PREVEND Study and the Gubbio Study reported data not only on kidney function change over time but also on mortality and causes of death.

## 3. Dietary Protein Intake: Assessments and Descriptive Statistics

The protein intake of persons on their own diet under free-living conditions can be assessed by two different methods: The collection of reported information, such as food records and questionnaires; the measurement of urine markers as total nitrogen or urea nitrogen. The use of questionnaires—either self-compiled, or self-administered, or administered by trained or specialized personnel—as well as the use of food records inevitably implies the major bias of the gap between the reported information and the true intake. The pro of questionnaires and food records is that they give the possibility to collect information on the habitual intake and on the relative percentage of vegetal and animal proteins. Urine markers as nitrogen and urea nitrogen have been extensively used as objective indices of protein intake, because urea is the end-product of protein catabolism and appears in serum and urine soon after the dietary intake of protein [14,15]. There are two cons of urine markers: They are affected by the day-to-day intra-individual variability in protein intake and cannot differentiate the intake of vegetal proteins from the intake of animal proteins. The main pro of urine markers is that, at variance with questionnaires and food records, they are objective measurable indices and can be easily converted to protein intake by a simple multiplier.

Dietary protein intake was assessed by questionnaires in the Nurses’ Health study and by urine urea nitrogen in the PREVEND study and the Gubbio Study. The estimates of protein intake averaged about 75 g/day in the Nurses’ Health study, 62 g/day in the PREVEND study, and 66 g/day in the Gubbio study. The inter-individual variability of protein intake was large in all studies. Protein intake ranged from less than 20 g/day to more than 130 g/day in the Nurses’ Health study that focused on habitual intake. The coefficient of variation, assessed as the standard deviation/mean ratio, was above 13% in all studies. Only the PREVEND study and the Gubbio study reported data about protein intake per kg of ideal weight that averaged the identical value of 1.2 g/day in both studies.

Overall, data about protein intake of the three studies substantially agreed. On average, in all studies, protein intake was higher than 60 g/d and higher that the minimum requirement, as expected in individuals living in industrialized countries. Nevertheless, in all studies, protein intake below the minimum requirement was found in a sizeable number of individuals, even when data collection targeted habitual intake. As far as the differences among the studies, the lower mean of estimated intake in the PREVEND study could reflect the fact that urine urea nitrogen is approximately 10% lower than urine total nitrogen and thus slightly underestimates protein intake, a bias which was partially compensated in the Gubbio study by the use of overnight urine instead of 24-hour urine [16]. 

## 4. Kidney Function: Assessments and Descriptive Statistics

Since 2003 the international guidelines have recommended the use of the estimated glomerular filtration rate (eGFR) for the assessment of kidney function, because eGFR is more accurate than other non-invasive indices of kidney function and can be easily calculated by readily available variables as serum creatinine, sex, age, and ethnicity [17]. For eGFR calculation, the initial recommendations suggested the use of the Cockcroft and Gault formula or of the equation developed by the Modification of Diet in Renal Disease (MDRD). The Cockcroft & Gault formula is the less accurate method for eGFR calculation due to systematic biases associated with underweight, overweight, and age [18]. The MDRD equation eliminated the influence of these confounders but had low accuracy in the range of eGFR ≥ 60 mL/min x 1.73 m^2^, because it was developed in a cohort of nephropathic patients where only a few individuals had eGFR values in the normal-high range [19]. Nowadays, the current guidelines recommend that the standard method for eGFR calculation should be the equation developed in 2009 by the Chronic Kidney Disease Epidemiology Collaboration (CKD-Epi) study [20]. This equation solved the problem of accuracy for eGFR ≥60 mL/min × 1.73 m^2^ having been developed in a large cohort, including both patients and healthy individuals of the general population. 

eGFR at the first exam (baseline) averaged 93 mL/min × 1.73 m^2^ in the Nurses’ Health study (MDRD equation), 81 mL/min × 1.73 m^2^ in the PREVEND study (MDRD equation), and 86 mL/min × 1.73 m^2^ in the Gubbio study (CKD-Epi equation). The prevalence of low kidney function, defined as eGFR <60 mL/min × 1.73 m^2^, was not reported in the Nurses’ Health study whereas was 5.9% and 5.8% in the PREVEND study and the Gubbio study, respectively. After normalization for the duration of the interval, from baseline exam to follow-up exam, eGFR change per year averaged −1.19 mL/min × 1.73 m^2^ in the Nurses’ Health study, −0.45 mL/min × 1.73 m^2^ in the PREVEND study, and −0.74 mL/min × 1.73 m^2^ in the Gubbio Study. 

## 5. Mortality: Assessments and Descriptive Statistics

As stated above, only the PREVEND Study and the Gubbio Study reported data on mortality. In both studies, data derived from death certificates and causes of death were coded by the International Classification of Diseases. The total number of deaths was 443 in the PREVEND study and 871 in the Gubbio study. Non-cardiovascular disease accounted for 69% and 52% of these events, respectively.

## 6. Relation of Protein Intake to Kidney Function Decline

**Figure 2:**
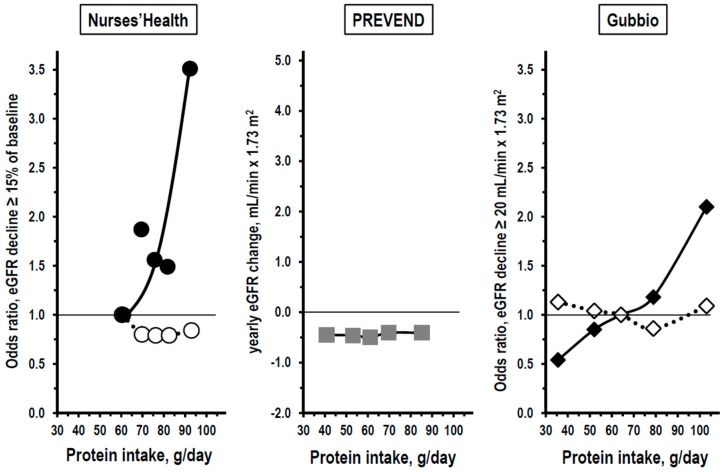
Relationships of baseline protein intake to kidney function decline in multivariate quintile analyses of the Nurses’ Health study, the Prevention of Renal and Vascular End-stage Disease (PREVEND) study, and the Gubbio study. Protein intake was assessed by a questionnaire in the Nurses’ Health study and by urine urea nitrogen in the PREVEND study and in the Gubbio study. Kidney function was assessed as estimated glomerular filtration rate (eGFR) in all studies. Outcomes of interest in vertical axis differed among the studies as follows: The Nurses’ Health study targeted the incidence at the second visit of eGFR decline ≥15% of baseline eGFR (left panel, vertical axis = odds ratio versus quintile 1, thin horizontal line = quintile 1 as reference); the PREVEND study targeted the mean annual change in eGFR from first visit to second visit (central panel, thin horizontal line = no change in eGFR); the Gubbio study targeted the incidence of eGFR decline ≥20 mL/min × 1.73 m^2^ below baseline eGFR (right panel, thin line = quintile 3 as reference). Open symbols are for the subgroup with non-reduced baseline eGFR (≥80 mL/min × 1.73 m^2^ in the Nurses’ Health study and ≥90 mL/min × 1.73 m^2^ in the Gubbio study); closed black symbols are for the subgroup with reduced baseline eGFR (<80 mL/min × 1.73 m^2^ in the Nurses’ Health study and <90 mL/min × 1.73 m^2^ in the Gubbio study); closed gray symbols are for data in the whole study cohort.

The results of the Nurses’ Health study, the PREVEND study, and the Gubbio study are comparable, because all of them included analyses by quintiles of baseline protein intake. In the Nurses’ Health study, primary outcomes of interest were an eGFR decrease from baseline exam to follow-up exam of at least 15%, 20%, or 25% of baseline eGFR. The conclusion of the Nurses’ Health study was that an association of protein intake with kidney function change over time was not detectable in the whole sample but was present only among women who had already at baseline mildly reduced kidney function. The left panel of Figure 2 shows in fact that the association between the baseline protein intake and the incidence of eGFR decrease ≥15% was flat among women with baseline eGFR ≥80 mL/min × 1.73 m^2^ but had a linear steep trend among women with baseline eGFR <80 mL/min × 1.73 m^2^. 

The trend was mainly due to the difference between quintile 1 and quintile 5 of protein intake and, in additional analyses, to the effect of high intake of non-dairy animal protein. In the PREVEND study, the outcome of interest was the yearly eGFR decline from baseline exam to follow-up exam. The central panel of Figure 2 shows that the association between the baseline protein intake and the yearly eGFR decline was flat as in women with baseline eGFR ≥80 mL/min × 1.73 m^2^ of the Nurses’ Health study. The authors of the PREVEND study did not include subgroup analyses nor analyses by stratum of baseline kidney function. Thus, they did not investigate whether an association of high protein intake with kidney function decline was actually detectable only in a subgroup of the population-sample. In the Gubbio study, the primary outcome of interest was an eGFR decrease from baseline exam to follow-up exam of at least 20 mL/min × 1.73 m^2^, a threshold that was derived from the standard deviation of the distribution of eGFR change over time in the Gubbio cohort. The conclusion of the Gubbio study was that an association of the baseline protein intake with kidney function decline was present only among men and women with reduced kidney function at baseline, a finding that confirmed the observation of the Nurses’ Health study in women with baseline eGFR <80 mL/min × 1.73 m^2^. The right panel of Figure 2 shows in fact that the association of baseline protein intake with the incidence of eGFR decrease ≥20 mL/min × 1.73 m^2^ was flat among persons with baseline eGFR ≥90 mL/min × 1.73 m^2^ but was linear and continuous among persons with baseline eGFR <90 mL/min × 1.73 m^2^, that is in the range of stage 2 chronic kidney disease. Both in the Nurses’ Health study and in the Gubbio study, findings were independent of sex, age, smoking, cardiovascular risk factors, and other confounders.

Despite the differences in methods assessments, time and place of exams, ethnicity, etc., the results of the three studies substantially agreed. An association of baseline protein intake with long term change over time in kidney function was absent, or at least non-detectable with the use of the currently available methods, in the general population and/or in persons with normal kidney function. Vice versa, a direct association of baseline protein intake with risk of kidney function decline was apparent when the analysis was limited to the subgroup of persons with below-normal kidney function. 

## 7. Relation of Protein Intake to Mortality

**Figure 3:**
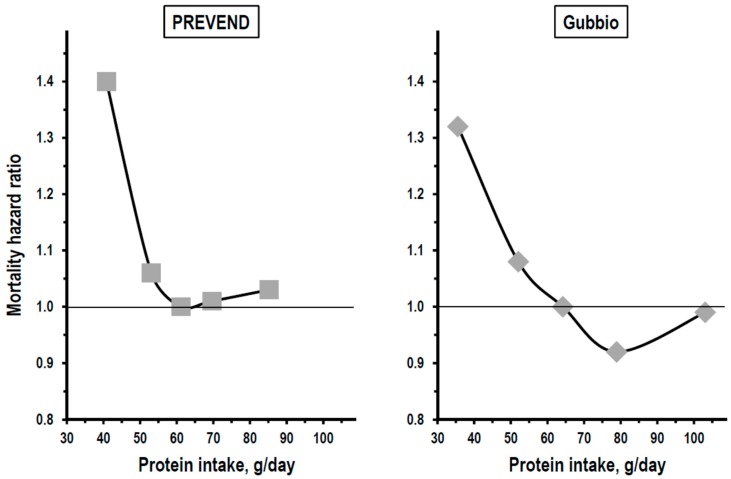
Relationships of baseline protein intake to long-term mortality in multivariate quintile analyses of the PREVEND study and the Gubbio study. Protein intake was assessed by urine urea nitrogen in both studies. Outcome of interest in vertical axis was the hazard ratio of all-cause mortality in both studies (thin line = mortality in quintile 3 as reference). Gray symbols are for data in the whole study cohort.

As for kidney function change, an analysis by quintile of baseline protein intake was a key statistical procedure both in the PREVEND study and in the Gubbio study. Based on the results of this analysis, both studies reported a non-linear J-curve relationship between protein intake and mortality. Data indicated in fact a risk excess of approximately 35% in all-cause mortality among persons with baseline protein intake in quintile 1, no association between protein intake and mortality in intermediate quintiles, and a weak, insignificant trend toward higher mortality among persons with baseline protein intake in quintile 5 (Figure 3). 

With data expression per kg of ideal weight, protein intake of quintile 1 averaged 0.61 g/day in the PREVEND study and 0.62 g/day in the Gubbio study indicating a consistent association with higher long-term mortality of protein intakes <0.8 g/kg of ideal weight, that is of intakes below the threshold recommended by the World Health Organization as a minimum requirement [1]. The mortality excess in quintile 1 of protein intake was detectable also after 10-year follow-up excluding the possibility that protein intake had been reduced secondary to disease(s) present already at baseline (13). In both studies, findings were independent of sex, age, smoking, cardiovascular risk factors, and other confounders. The mortality excess associated with low protein intake was independent of baseline kidney function also and, more importantly, was consistent in persons with eGFR ≥90 mL/min × 1.73 m^2^ and in persons with eGFR <90 mL/min × 1.73 m^2^ [13]. Findings of the PREVEND study and the Gubbio study were consistent also with regard to causes of death because non-cardiovascular mortality accounted for the mortality excess associated with low protein intake in both studies. Moreover, a multi-variable competing-risks regression analysis of the Gubbio study indicated a 33% excess risk of death for neoplastic disease in persons with protein intake in the lowest quintile.

## 8. Conclusions

Low protein diets have been for years a therapeutic intervention to slow down the progression of chronic kidney disease toward kidney failure. However, consistent long-term research data are missing on the efficacy and safety of low protein diets. Epidemiological observational data indicate that, only in persons with kidney function below the optimal level, the level of protein intake is linearly related to the incidence of the decline of kidney function over time. Given the association of protein intake with mortality also, a competing risk analysis should be the method for investigation about the possible effects of dietary protein on kidney function. In the sole study with relevant data, non-dairy animal proteins accounted for this effect more than vegetal proteins. Thus, the epidemiological evidence supports the view that high protein intake could be an unhealthy habit in persons with kidney disease, even in the early stages of the disease when kidney function is only slightly reduced. Cause-effect relationships are difficult to assess in observational studies. However, with regard to possible mechanisms linking high protein intake to kidney function decline, epidemiological data indicate also a cross-sectional association of high protein intake with high eGFR [21]. Thus, the epidemiological evidence is in agreement also with the hypothesis that chronic or repeated up-regulation of glomerular filtration secondary to high protein intake could be one of the factors hastening the progression of kidney disease toward kidney failure [2,3,4].

On the other hand, epidemiological data do not allay and actually reinforce the doubts that a chronic reduction in protein intake below the minimum requirement could be dangerous for overall health. Findings of population-based studies performed in two European countries with different dietary habits—the first one in northern-central Europe and the second one in the Mediterranean area —indicate in fact that persons with protein intake <0.8 g/day per kg of ideal weight have an increased long-term risk of mortality mainly due to an excess risk of non-cardiovascular disease. Similar observations were reported by clinical studies also in kidney transplant and non-renal-diseases [22,23,24,25]. Both the available population-based studies suggest that no excess risk was detectable when protein intake ranges above the threshold of 0.8 g/day per kg of ideal weight, that is above the threshold recommended by the World Health Organization for prevention of protein malnutrition. With regard to mechanisms, the association of low protein intake with a mortality excess for non-cardiovascular diseases could reflect a protective effect against these diseases of protein-rich vegetable foods [26,27]. Other possibilities cannot be excluded.

Altogether, epidemiological evidence indicates two important relationships of the level of protein intake with health: A linear relation to the long-term risk of kidney function decline limited to persons with below-normal kidney function; a non-linear relation to the long-term risk of mortality in the presence and in the absence of kidney dysfunction. One limitation of this review was the lack of a meta-analysis of individual data that would represent the best method to investigate these two relationships. Nevertheless, the combination of the two relationships suggests that, in persons with progressive kidney disease, protein intake should be restricted to limit the kidney function decline but should not approach the levels associated with long-term risk of mortality. In this view, the minimum requirement recommended by the World Health Organization of 0.8 g/day of protein per kg of ideal weight appears as a reasonable dietary regimen theoretically capable to avoid not only the acceleration of kidney function decline over time, due to high protein intake but also non-renal risks, due to low protein intake. This dietary regimen should include more vegetal protein than animal protein considering the evidence of more untoward effects of animal protein. A diet with 0.8 g/day of protein per kg of ideal weight could hardly be defined as low-protein although it would represent in the large majority of persons a substantial reduction of their habitual protein intake. As a matter of fact, protein intake ranged well above that level in most of the examinees of all epidemiological studies. Regardless of kidney dysfunction, the epidemiological evidence supports the recommendation that protein intake should not be lower than 0.8 g/day per kg of ideal weight. Unless new research data solve the doubts about the safety of low protein diets, physicians and patients should be aware that long-term intake of protein below that threshold could imply severe unfavourable effects for the health.
